# Supplementary material for: Opening gap width influences distal tibial rotation below the osteotomy site following open wedge high tibial osteotomy
Source: PLoS One. 2020 Jan 16;15(1):e0227969. doi: 10.1371/journal.pone.0227969 (PMC6964860; doi:10.1371/journal.pone.0227969)
Supplement: Attachment — Submitted filename: Reply to reviewers Revision.docx [file pone.0227969.s002.docx]

Dear Editor,

Thank you for your kind consideration regarding our paper.

We revised our manuscript according to the reviewers’ comments. Details of our responses are below and changes in the manuscript are marked in red.

We look forward to hearing from you regarding this paper.

Reply to reviewer’s comments

Reviewer #1: Q1. Regarding the hinge, you described that efforts were made to keep the lateral cortex and lateral capsular hinge intact (lines 94 to 95). However, the actual hinge situation was not reported. The rate of hinge fracture is said to be at least over 20% when evaluated CT scans. Fortunately, you have all CT data for making the 3D analysis. **Could you examine the presence or absence of the hinge fracture and the type of the fracture according to Takeuchi classification?** As you mentioned, large opening gap tend to make unstable hinge fracture. **If the rate of hinge fracture was high in the large opening gap group than in the small opening gap group, hinge fracture could be the predictor of the distal tibial rotation**.

Author’s response: Thanks for your comment. We examined the presence or absence of the lateral hinge fracture and the type of the hinge fracture based on Takeuchi classification. (Line 209-210) We compared the rate of lateral hinge fracture between two groups. (Table 2) Also, we compared the opening gap width between the presence and absence of lateral hinge fractures. The opening gap width was 9.94 ± 3.20 and 11.13 ± 1.85 in the absent and present of lateral hinge fractures, respectively, however there was no significant difference (P = 0.192). (This result was not added in the revised manuscript because it was out of our study’s scope and insignificant result. If you want to put in the manuscript, we will do it.)

- Postoperative CT scans showed that 15 of the 42 knees had lateral hinge fractures. All 15 were classified as type I, with none classified as type II or III.
- **Table 2.** Demographic data, opening gap, and preoperative and postoperative alignment in knees with rotation greater than 3° and less than 3°

|  | Dital tibial rotation ≤ 3°  (n=24) | Dital tibial rotation > 3°  (n=18) | P-value |
| --- | --- | --- | --- |
| Age | 57.1 ± 6.9 | 56.2 ± 7.4 | 0.669 |
| BMI | 27.4 ± 3.2 | 27.7 ± 3.4 | 0.791 |
| Opening gap with | 9.6 ± 2.9 | 11.4 ± 2.4 | 0.027 |
| Preoperative MA | 6.0 ± 3.0 | 7.6 ± 2.5 | 0.078 |
| Postoperative MA | -3.4 ± 2.5 | -3.3 ± 2.0 | 0.833 |
| Alignment correction | 9.5 ± 3.9 | 10.9 ± 3.2 | 0.192 |
| Lateral hinge fractures | 9 | 6 | 0.780 |
| Angle of vertical osteotomy direction | 23.1 ± 8.6 | 28.3 ± 7.6 | 0.051 |
| Biplane osteotomy angle | 105.1 ± 9.4 | 107.1 ± 8.4 | 0.468 |

BMI, body mass index; MA, mechanical axis

Q2. You compared the demographic data of the two groups (distal tibial rotation <3 degrees and distal tibial rotation>3 degrees) including opening gap width, preoperative MA, postoperative MA, and alignment correction (Table 2). However, the alignment correction is estimated by the subtraction of the preoperative MA from the postoperative MA. Furthermore, the alignment correction would be calculated by the opening gap and the tibial width. Therefore, these parameters have too many confounding biases each other. On the contrary, **there would be more parameters which could have some relationships with tibial rotation; such as the angle between the ascending cut and the transverse cut, the direction of the ascending cut on the axial slice, or lateral hinge fracture**, and so on. **Reconsider the necessary parameters to be compared in the both groups.20:** What is the clinical relevance?

The journal now requires “Level of Evidence”. This information should be added at the end of the abstract. This must be given for all clinical studies. Please read and follow the information found in “Instructions to Authors” for the level of evidence.

Author’s response: Thanks for your sincere comments. We really appreciated your careful consideration of our study. Thanks to your comments, we could thoroughly consider about new variables that could have affected the tibial rotation after high tibial osteotomy. We added new variables, such as lateral hinge fracture, the angle of vertical osteotomy direction, and biplane osteotomy angle.

- CT Analysis

All CT data were obtained by the same CT scanner (Lightspeed VCT; GE Medical Systems, Milwaukee, WI). The collimation was 16 x 0.625 mm, the slice thickness was 0.625 mm, and the acquisition matrix was 512 x 512. Postoperative CT scan was performed on postoperative day 5 after removing the hemovac drain. Lateral hinge fracture classified by Takeuchi’s method was detected by thorough investigation of coronal images. The direction of vertical osteotomy was determined by the angle between vertical osteotomy line and posterior margin of tibia plateau on axial images (Fig. 1a,b). The angle of biplane osteotomy was evaluated between vertical and oblique osteotomy at the level of tibial tuberosity on sagittal images (Fig. 1c). (Line 116-125)

- (Figure 1)
- **Fig. 1. Measurement of the angle of vertical osteotomy direction and biplane osteotomy angle on computed tomography. (A)** Line 1, a tangent line to posterior tibia plateau is drawn on the axial image of tibia plateau level. (B) Line 1’, copied and pasted from Fig. 1A. The angle of vertical osteotomy direction is determined between line 1’ and vertical osteotomy (line 2). (C) A sagittal image at the prominent tibial tuberosity level. The biplane osteotomy angle is determined between vertical osteotomy (line 3) and oblique osteotomy (line 4). (Line 126-131)
- **Table 2.** Demographic data, opening gap, and preoperative and postoperative alignment in knees with rotation greater than 3° and less than 3° (Line 224-226)

|  | Dital tibial rotation ≤ 3°  (n=24) | Dital tibial rotation > 3°  (n=18) | P-value |
| --- | --- | --- | --- |
| Age | 57.1 ± 6.9 | 56.2 ± 7.4 | 0.669 |
| BMI | 27.4 ± 3.2 | 27.7 ± 3.4 | 0.791 |
| Opening gap with | 9.6 ± 2.9 | 11.4 ± 2.4 | 0.027 |
| Preoperative MA | 6.0 ± 3.0 | 7.6 ± 2.5 | 0.078 |
| Postoperative MA | -3.4 ± 2.5 | -3.3 ± 2.0 | 0.833 |
| Alignment correction | 9.5 ± 3.9 | 10.9 ± 3.2 | 0.192 |
| Lateral hinge fractures | 9 | 6 | 0.780 |
| Angle of vertical osteotomy direction | 23.1 ± 8.6 | 28.3 ± 7.6 | 0.051 |
| Biplane osteotomy angle | 105.1 ± 9.4 | 107.1 ± 8.4 | 0.468 |

- The preoperative varus (*r* = 0.402, *P* = .028), angle of vertical osteotomy direction angle (r = 0.342, *P* = 0.027) and opening gap width (*r* = 0.294, *P* = .006) were associated with distal tibial rotation on correlation analysis (Table 3). (Line 228-233)

**Table 3.** Correlations between distal tibial rotation and pre- and postoperative alignment, opening gap, and alignment correction before and after opening wedge high tibial osteotomy

| Distal tibial rotation | | |
| --- | --- | --- |
|  | Correlation coefficient | P-value |
| Preoperative MA | 0.402 | 0.028 |
| Opening gap width | 0.418 | 0.006 |
| Postoperative MA | 0.075 | 0.636 |
| Alignment correction | 0.272 | 0.082 |
| Angle of vertical osteotomy direction | 0.342 | 0.027 |
| Biplane osteotomy angle | 0.199 | 0.207 |

- However, on multiple regression analysis, the opening gap width was the only predictor of distal tibial rotation (*β* = 0.418, *P* = .006, Table 4). (Line 235-240)

**Table 4.** Multiple linear regression analysis of factors affecting the change of the posterior slope after opening wedge HTO in all included subjects

| Dependent  Variables | Independent  Variables | Non-standardized Coefficients | | Standardized  Coefficients |  |
| --- | --- | --- | --- | --- | --- |
|  |  | B | SE | β | P-value |
| Distal tibial rotation | Age | -0.003 | 0.050 | -0.058 | 0.954 |
|  | Preoperative MA | 0.091 | 0.247 | 0.114 | 0.715 |
|  | Opening gap width | 0.341 | 0.117 | 0.418 | 0.006 |
|  | Postoperative MA | 0.265 | 0.245 | 0.250 | 0.286 |
|  | Alignment correction | -0.113 | 0.166 | -0.168 | 0.499 |
|  | Angle of vertical osteotomy direction | 0.069 | 0.040 | 0.253 | 0.094 |
|  | Biplane osteotomy angle | 0.048 | 0.039 | 0.184 | 0.229 |

Q3. As you mentioned in the discussion section (lines 243 to 246), hamstring tendon acts as an internal rotator. Therefore, how to treat the pes and MCL in OWHTO may affect the tibial rotation. **Could you describe more detailed procedure regarding medial soft tissues in the Surgical Technique section?** There seems to be some contradictions suggested in the following Q4.

Author’s response: Thanks for your valuable comment. We added more detailed surgical procedure regarding soft tissues in the method section.

- The patellar tendon was exposed for vertical osteotomy. After identifying the pes anserine tendon, the fascia overlying tendon was incised and release toward posterior capsule and pes anserine tendon was detached from the tibia. Also, the superficial medial collateral ligament (MCL) was identified and distal superficial MCL was carefully released. Then, posterior capsule was incised vertically along the posterior margin of tibia and was released from the tibia. (Line 87-92)

Q4. In your technique, the oblique osteotomy line, i.e. transverse cut, is started from 5 to 6 cm from the joint line. In general, about 4cm is recommended for the starting point.

If you cut from 5 to 6cm from the joint line,

1) The cutting line is far below from the attachment of the hamstring tendons. Therefore, the larger amount of the release in the pes would be required. It may create larger external rotation of the tibia.

2) You mentioned that the MCL distal insertion remained intact because the osteotomy was performed just distal to the insertion site of the MCL. However, the superficial MCL attaches 7 to 8 cm from the joint line. Therefore, when applying the 5 to 6cm cutting line, the MCL could be cut during the osteotomy.

Author’s response: Thanks for your comments. First of all, we are sincerely sorry about the wrong information in the surgical technique. The osteotomy level was 3 to 4 cm distal to joint line as usual methods and distal MCL was inevitably released in the distal insertion area. However, we fully released pes anserine tendon and additionally investigated on vertical osteotomy direction, which might be reasons for the external rotation of distal tibia. Again, we are very sorry about the mistakes.

- . The oblique osteotomy part of the biplane osteotomy was intended to commence at the medial tibial cortex along the metaphyseal flare (approximately 3 to 4 cm distal to the joint line) (Line 95-98)
- However, our technique was different from that used in the above two studies in that the hamstring tendon was detached completely from the tibial insertion site, and in most cases. The difference in hamstring management could explain in part the different direction and amount of distal tibial rotation; the internal rotation was relatively large (4° to 5°) in the previous two studies, and the external rotation was small (2.7°) in our study. Also, the direction of vertical osteotomy in axial plane might be reason for external rotation of distal tibia in the study. According to the current result, the vertical osteotomy was directed from anteromedial to posterolateral in axial plane, which might result in external rotation of distal tibia. Despite statistically insignificance, the group of dital tibial rotation > 3° tended to have larger angle of vertical osteotomy direction, representing more posterolateral direction possibly causing external rotation, than the group of distal tibial rotation≤ 3° (*P* = 0.051). (Line 272-282)
